# Commentary: Forgetting the best when predicting the worst: preliminary observations on neural circuit function in adolescent social anxiety

**DOI:** 10.3389/fpsyg.2018.01088

**Published:** 2018-07-11

**Authors:** Rodrigo S. Fernández, María E. Pedreira, Mariano M. Boccia, Laura Kaczer

**Affiliations:** ^1^Laboratorio de Neurobiología de la Memoria, Departamento de Fisiología y Biología Molecular y Celular, Instituto de Fisiología, Biología Molecular y Neurociencias-CONICET, Buenos Aires, Argentina; ^2^Facultad de Ciencias Exactas y Naturales, Universidad de Buenos Aires, Buenos Aires, Argentina; ^3^Laboratorio de Neurofarmacología de los Procesos de Memoria, Cátedra de Farmacología, Facultad de Farmacia y Bioquímica, Universidad de Buenos Aires, Buenos Aires, Argentina

**Keywords:** anxiety disorders, memory reconsolidation, prediction error, social anxiety, adolescent

Let's imagine someone who is about to perform a presentation at work. As he enters the meeting, he feels self-conscious that he has drawn attention to himself. He thinks “*this could be a disaster, I must do it perfectly*.” His heart-rate speeds up and he begins to sweat. However, he manages to finish the presentation successfully and he receives positive comments from his colleagues. The following day, he recalls: “*the presentation went terrible*.”

The aforementioned situation is not unusual. Social anxiety (SA) is one of the most prevalent forms of anxiety (Costello et al., 2005). Individuals with SA fear negative evaluation and persistently avoid social situation (Stein and Stein, 2008; American Psychiatric Association, 2013). This causes them marked disability such as; experience difficulty communicating, eating and talking in public, and negatively impacts their social functioning (Liebowitz, 1987; Hazen and Stein, 1995). SA typically begins before the end of adolescence, when increased complexity and salience of peer relationships requires novel forms of social learning (Brown and Larson, 2009; Crone and Dahl, 2012). Importantly, when untreated, this disorder tend to be the most persistent of anxiety disorders (Stein and Stein, 2008). Thus, one of the most challenging endeavors is to understand the mechanisms involved in SA maintenance. Several lines of research highlighted the alteration of different cognitive and learning processes (Foa et al., 1996; Clark and Beck, 2011). According to one popular model, proposed by Clark and Wells (1995), SA persists due to a shift of the attention focus to internal cues, the use of internal cues to interpret how one appears to others and the use of safety behaviors/avoidance. Other models highlighted the role of negative self-images and aversive memories, probably rooted in early experiences during development (Hirsch et al., 2003; Moscovitch et al., 2011).

Prediction error (PE) is defined as a mismatch or an incongruence between predicted and occurred events and it has been proposed as the driving force of learning (Rescorla and Wagner, 1972; Fernández et al., 2016). Recently, it has been proposed that an altered PE signaling during adolescence may contribute to some of the mechanisms that help to maintain SA (Pfeifer et al., 2013; Nelson et al., 2014). Furthermore, dysfunctional PE processing could lead to deficient recall of positive social experiences, which in turn could promote negative social expectations and interpretation biases, frequently observed in SA (Clark and McManus, 2002). Thus, it is of interest to address the implication of alterations in PE signaling during “*post-mortem*” processing in the maintenance of mental disorders.

A recent study by Jarcho and colleagues (Jarcho et al., 2015) analyzed the relationship between PE and social learning, comparing socially anxious adolescents and non-anxious adolescents, as well as adults. Using a social learning task (“Chatroom Task” Guyer et al., 2009), participants are led to believe they would chat online with a peer predicted, then received, social feedback from high and low-value peers. Later, participants recall the social feedback they received from each peer. Neural correlates of social evaluation were assessed by fMRI scanning, comparing engagement to expected and unexpected positive and negative feedback (PE). Results showed that for socially anxious adolescents, but not adults or controls of either age group, there was an impairment in memory for social feedback. That is, when socially anxious adolescents predicted that someone would not chat with them, but the feedback contradicted this prediction (i.e., positive PE), there is an impaired recall of this event. The authors reported that this memory impairment was correlated with a negative fronto-striatal functional connectivity, suggesting a dysregulated PE signaling in socially anxious adolescents. These results point to a deficit in memory updating, as these participants were not able to change their negative expectations regarding social feedback when the outcome was better than expected. We suggest that these findings could also be analyzed and/or interpreted from the memory reconsolidation perspective, providing complementary tools to address the mechanisms of socially anxiety maintenance.

Reconsolidation is the mechanism that allows consolidated memories to be updated (Dudai, 2012; Fernández et al., 2016). Thus, our brain is able to update its stored representations in content, strength, and/or expectations by this process (Lee, 2009). However, certain memory features such as the age and strength constrain memory reconsolidation (Fernández et al., 2016). Interestingly, only when there is a mismatch between what is expected and what actually occurs (PE), a reactivated memory enters in a transient labile state (destabilization) followed by its restabilization in order to persist (reconsolidation) (Dudai, 2012; Exton-McGuinness et al., 2014; Fernández et al., 2016; Beckers and Kindt, 2017). Recently, Fernández et al. (2017) postulated a theoretical framework for how anxiety disorders are maintained through impaired memory updating due to a dysfunctional PE minimization strategy. We suggest that this framework could be useful to discuss the results of Jarcho and coworkers (Jarcho et al., 2015). Specifically, why people suffering from SA cannot modify their negative predictions in the light of disconfirmatory evidence (i.e., receiving positive feedback)? Why the positive feedback is not even recalled? In this context, the repeated violation of expectations (PE) should destabilize and re-stabilize memory (update prior predictions) with new safety information (Salkovskis, 1991). However, none of this occurs and dysfunctional memories are maintained or strengthened. What is inside the core of anxiety that prevents memory updating? In highly anxious individuals, when strong and precise memories encounter a PE, the destabilization phase of reconsolidation begins. During the restabilization phase, the error generated, that would otherwise force memory content updating (schema re-organization), is affected. Moreover, PE minimization is accomplished by assimilation (Fernández et al., 2017; Gilboa and Marlatte, 2017) in accordance to prior belief facilitated by the altered cognitive and attentional processes such as those proposed by Clark and Wells (1995). During the “*post-mortem*” processing, the prediction generated by a strong memory enters in a self-confirmatory vicious cycle (Clark and Beck, 2011) leading to a “blindness” to incongruent information (Fernández et al., 2017). Hence, strong top-down modulation affects experience and in consequence the original prediction and the strong memory persist. In a sense, this strong memory acts as boundary condition for the reconsolidation process. One could speculate that this deficit could be mediated by the negative fronto-striatal functional connectivity and probably the basolateral-amygdala. Albeit Jarcho et al. (Nelson et al., 2014) did not report amygdala activation, this structure plays a key role in aversive memory-updating, and it is known to be hyperactive in anxiety disorders and particularly in social preference processing in SA (Grupe and Nitschke, 2013; Blair et al., 2016).

Currently there is a strong evidence supporting the role of cognitive process, attentional shifting and safety seeking behaviors, in SA maintenance (Grupe and Nitschke, 2013). Here we highlighted the findings of Jarcho et. al and proposed an underlying mechanism (memory reconsolidation) for SA persistence. Altered memory reconsolidation could prevent the incorporation of incongruent information (memory updating), and perpetuate the dysfunctional memory.

## Author contributions

Conceived and wrote the paper: RF, MP, MB, and LK.

### Conflict of interest statement

The authors declare that the research was conducted in the absence of any commercial or financial relationships that could be construed as a potential conflict of interest.
